# Automated mood disorder symptoms monitoring from multivariate time-series sensory data: getting the full picture beyond a single number

**DOI:** 10.1038/s41398-024-02876-1

**Published:** 2024-03-26

**Authors:** Filippo Corponi, Bryan M. Li, Gerard Anmella, Ariadna Mas, Isabella Pacchiarotti, Marc Valentí, Iria Grande, Antoni Benabarre, Marina Garriga, Eduard Vieta, Stephen M. Lawrie, Heather C. Whalley, Diego Hidalgo-Mazzei, Antonio Vergari

**Affiliations:** 1 School of Informatics, University of Edinburgh, Edinburgh, UK; 2 Bipolar and Depressive Disorders Unit, Department of Psychiatry and Psychology, Hospital Clínic de Barcelona, c. Villarroel, 170, 08036 Barcelona, Spain; 3 Institut d’Investigacions Biomèdiques August Pi i Sunyer (IDIBAPS), c. Villarroel, 170, 08036 Barcelona, Spain; 4 Centro de Investigación Biomédica en Red de Salud Mental (CIBERSAM), Instituto de Salud Carlos III, Madrid, Spain; 5 Departament de Medicina, Facultat de Medicina i Ciències de la Salut, Universitat de Barcelona (UB), c. Casanova, 143, 08036 Barcelona, Spain; 6 Division of Psychiatry, Centre for Clinical Brain Sciences, University of Edinburgh, Edinburgh, UK; 7 Generation Scotland, Institute for Genetics and Cancer, University of Edinburgh, Edinburgh, UK

**Keywords:** Biomarkers, Diagnostic markers

## Abstract

Mood disorders (MDs) are among the leading causes of disease burden worldwide. Limited specialized care availability remains a major bottleneck thus hindering pre-emptive interventions. MDs manifest with changes in mood, sleep, and motor activity, observable in ecological physiological recordings thanks to recent advances in wearable technology. Therefore, near-continuous and passive collection of physiological data from wearables in daily life, analyzable with machine learning (ML), could mitigate this problem, bringing MDs monitoring outside the clinician’s office. Previous works predict a single label, either the disease state or a psychometric scale total score. However, clinical practice suggests that the same label may underlie different symptom profiles, requiring specific treatments. Here we bridge this gap by proposing a new task: inferring all items in HDRS and YMRS, the two most widely used standardized scales for assessing MDs symptoms, using physiological data from wearables. To that end, we develop a deep learning pipeline to score the symptoms of a large cohort of MD patients and show that agreement between predictions and assessments by an expert clinician is clinically significant (quadratic Cohen’s κ and macro-average F1 score both of 0.609). While doing so, we investigate several solutions to the ML challenges associated with this task, including multi-task learning, class imbalance, ordinal target variables, and subject-invariant representations. Lastly, we illustrate the importance of testing on out-of-distribution samples.

## Introduction

Mood disorders (MDs) are a group of diagnoses in the Diagnostic and Statistical Manual 5th edition [1] (DSM-5) classification system. They are a leading cause of disability worldwide [2] with an estimated total economic cost greater than USD 326.2 billion in the United States alone [3]. They encompass a variety of symptom combinations affecting mood, motor activity, sleep, and cognition and manifest in episodes categorized as major depressive episodes (MDEs), featuring feelings of sadness and loss of interest, or, at the opposite extreme, (hypo)manic episodes (MEs), with increased activity and self-esteem, reduced need for sleep, expansive mood and behavior. As per the DSM-5 nosography, MDEs straddle two nosographic constructs, i.e., Major Depressive Disorder (MDD) and Bipolar Disorder (BD), whereas MEs are the earmark of BD only [4].

Clinical trials in psychiatry to this day entirely rely on clinician-administered standardized questionnaires for assessing symptoms’ severity and, accordingly, setting outcome criteria. With reference to MDs, Hamilton Depression Rating Scale-17 [5] (HDRS) and Young Mania Rating Scale [6] (YMRS) are among the most widely used scales to assess depressive and manic symptoms [7], quantifying behavioral patterns such as disturbances in mood, sleep, and anomalous motor activity. The low availability of specialized care for MDs, with rising demand straining current capacity [8], is a major barrier to this classical approach to symptom monitoring. This results in long waits for appointments and reduced scope for pre-emptive interventions. Current advances in machine learning (ML) [9] and the widespread adoption of increasingly miniaturized and powerful wearable devices offer the opportunity for personal sensing, which could help mitigate the above problems [10]. This can involve a near-continuous and passive collection of data from sensors, with the aim of identifying digital biomarkers associated with mental health symptoms at the individual level, therefore backing up clinical evaluation with objective and measurable physiological data. Personal sensing holds great potential for being translated into clinical decision support systems [11] for the detection and monitoring of MDs. Specifically, it could be particularly appealing to automate the prediction of the items of the HDRS and YMRS scales as they correlate with changes in physiological parameters, conveniently measurable with wearable sensors [12–14].

**Fig. 1:**
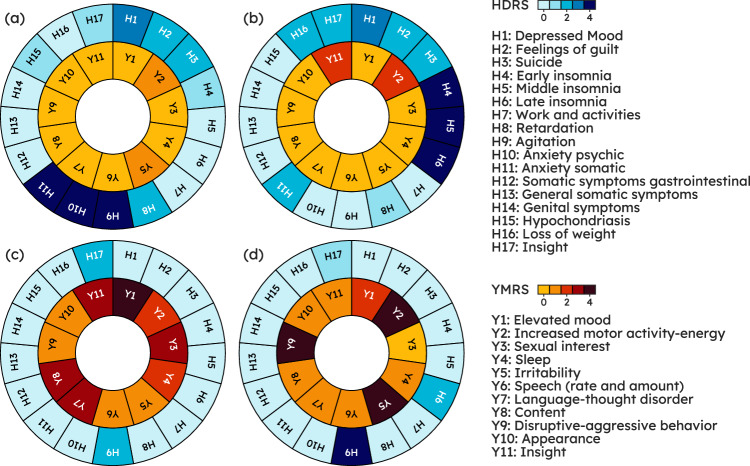
The same severity level can be realized from different symptom combinations, underlying different treatment needs. Top row: a pair of patients with Major Depressive Disorder on a Major Depressive episode; while both share the same severity levels, total Hamilton Depression Rating Scale (HDRS) ≥ 23 [33]. Patient (**a**), with total HDRS = 24, exhibits high levels of anxiety (H9, H10, H11), whereas patient (**b**), with total HDRS = 26, displays a marked insomnia component (H4, H5, H6). Bottom row: a pair of patients with Bipolar Disorder on a Manic Episode with a total Young Mania Rating Scale (YMRS) ≥ 25. Patient (**c**), with total YMRS = 30, has an irritable/aggressive profile (Y2, Y5, Y9) whereas patient (**d**), with total YMRS = 30, has a prominently elated/expansive presentation (Y1, Y3, Y7, Y11). Knowing what specific symptoms underlie a given state may allow clinicians to tailor treatment accordingly: e.g., a molecule with a stronger anxiolytic profile such as paroxetine or a short course of a benzodiazepine as an antidepressant is introduced may be appropriate in patient (**a**) whereas patient (**b**) might benefit from a compound with marked hypnotic properties such as mirtazapine.

However, so far, the typical approach has been to reduce MDs detection to the prediction of a single label, either the disease state or a psychometric scale total score [15, 16], which risks oversimplifying a much more complex clinical picture. Figure 1 illustrates this issue: patients with different symptoms and thus (potentially very) different scores on individual HDRS and YMRS items are “binned together” in the same category, leading to a loss of actionable clinical information. Predicting all items in these scales can instead align with everyday psychiatric practice where the specialist, when recommending a given intervention, considers the specific features of a patient, including their symptom patterns, beyond a reductionist disease label [17, 18]. Figure 1 illustrates a case in point where knowledge of the full symptom profile might enable bespoke treatment: on the face of it, patient (a) and (b) (top row) share the same diagnosis, i.e., MDE in the context of MDD; however, considering their specific symptom profile patient (a) might benefit from a molecule with stronger anxiolytic properties whereas patient (b) might require a compound with hypnotic properties. Furthermore, an item-wise analysis can lead to the identification of drug symptom specificity in clinical trials [19, 20].

Table 1 summarizes previous works in personal sensing for MDs and shows that all previous tasks collapsed the complexity of MDs to a single number. Côté-Allard et al. [21] explored a binary classification task, that is distinguishing subjects with BD on an ME from different subjects with BD recruited outside of a disease episode, when stable. The study experimented with different subsets of pre-designed features from wristband data and proposed a pipeline leveraging features extracted from both short and long segments taken within 20-hour sequences.

**Table 1:** This work is the first in personal sensing for MDs attempting to infer the full symptom profile, providing actionable clinical information beyond a single reductionist label, and it also stands out for the relatively large sample size (the largest among studies where MD acute phase clinical evaluation was not retrospective).

	Device(s)	Num. Patients	Patients Features	Task
This work	Empatica E4	75	MDD, BD; M_age_ = 44.16 SD_age_ = 14.42 F_%_ = 56	HDRS and YMRS items multi-task regression
Côté-Allard et al. [21]	Empatica E4	47	BD; M_age_ = 44 SD_age_ = 15 F_%_ = 67.24	Mania vs Euthymia binary classification
Ghandeharioun et al. [23]	Empatica E4 and Android Phone	12	MDD; M_age_ = 37 SD_age_ = 17 F_%_ = 75	HDRS total score regression
Pedrelli et al. [22]	Empatica E4 and Smartphone	31	MDD; M_age_ = 33.7 SD_age_ = 14 F_%_ = 74	HDRS total score regression
Jacobson et al. [25]	Actiwatch	23	MDD; M_age_ = 48.2 SD_age_ = 11.0 F_%_ = 43	Depression detection binary classification
Tazawa et al. [24]	Silmee W20	45	MDD, BD; M_age_ = 52.1 SD_age_ = 13.2 F_%_ = 46.7	Depression detection binary classification
Nguyen et al. [26]	Actiwatch	45	MDD, SCZ; M_age_ = 44.70 SD_age_ = 11 F_%_ = 73.33	Disease detection binary/multi-class classification
Lee et al. [27]	Fitbit Charge Hr 2 or 3 and Smartphone	270	MDD, BD; M_age_ = 23.3 SD_age_ = 3.63 F_%_ = 54.5	Mood episode prediction binary classification

Previous studies recruiting patients with either a DSM or an International Classification of Diseases (ICD) MD diagnosis and using passively collected wearable data are reported. F_%_: Percent Females; M_age_: mean age; SD_age_: standard deviation age.

Pedrelli et al. [22], expanding on Ghandeharioun et al. [23], used pre-designed features from a wristband and a smartphone to infer HDRS residualized total score (that is total score at time *t* minus baseline total score) with traditional ML models. Tawaza et al. [24] employed gradient boosting with pre-designed features from wristband data and pursued case-control detection in MDD and, secondarily, HDRS total score prediction. Similarly, Jacobson et al. [25] predicted case-control status in MDD from actigraphy features with gradient boosting. Nguyen et al. [26] used a sample including patients with either schizophrenia (SCZ) or MDD wearing an actigraphic device and explored case-control detection where SCZ and MDD were either considered jointly (binary classification) or as separate classes (multi-class classification). Of notice, this was the first work to apply artificial neural networks (ANNs) directly on minimally processed data, showing that they outperformed traditional ML models. Lastly, the multi-center study of Lee et al. [27] investigated mood episode prediction with a random forest and pre-designed features from wearable and smartphone data. Further to proposing a new task, our work stands out for a sample size larger than all previous works by over 2 dozen patients, with the exception of a multi-center study by Lee et al. [27], where, however, clinical evaluation was carried out retrospectively, thereby inflating chances of recall bias [28] and missing out on the real-time clinical characterization of the acute phase. Indeed, collecting data from patients on an acute episode, using specialist assessments and research-grade wearables, is a challenging and expensive enterprise. Relatively to previous endeavors, the contribution of this work is two-fold: (1) Taking one step beyond the prediction of a single label, which misses actionable clinical information, we propose a new task in the context of MDs monitoring with physiological data from wearables: inferring all items in HDRS (17 items) and YMRS (11 items), as scored by a clinician, which enables a fine-grained appreciation of patients’ psychopathology therefore creating opportunity for tailored treatment (Fig. 1). (2) We investigate some of the methodological *challenges* associated with the task at hand and explore possible ML solutions. **c1**: inferring multiple target variables (28 items from two psychometric scales), i.e., multi-task learning (MTL, see Section 3.4.1). **c2**: modeling ordinal data, such are HDRS and YMRS items (see Section 3.4.1). **c3**: learning subject-invariant representations, since, especially with noisy data and sample size in the order of dozens, models tend to exploit subject-idiosyncratic features rather than learning disease-specific features shared across subjects, leading to poor generalization [29] (see Section 3.4.2). **c4**: learning with imbalanced classes, as patients on an acute episode usually receive intensive treatment and acute states therefore tend to be relatively short periods in the overall disease course [30, 31] thereby tilting items towards lower ranks.

## Methods

### Data collection and cohort statistics

The following analyses are based on an original dataset, TIMEBASE/INTREPIBD, being collected as part of a prospective, exploratory, observational, single-center, longitudinal study with a fully pragmatic design embedded into current real-world clinical practice. A detailed description of the cohort is provided in Anmella et al. [32]. In brief, subjects with a DSM-5 MD diagnosis (either MDD or BD) were eligible for enrollment. Those recruited on an acute episode had up to four assessments: **T0** acute phase (upon hospital admission or at the home treatment unit), **T1** response onset (50% reduction in total HDRS/YMRS), **T2** remission (total HDRS/YMRS ≤7), and **T3** recovery (total HDRS/YMRS continuously ≤7 for a period of ≥8 weeks) [33]. On the other hand, subjects with a historical diagnosis but clinically stable at the moment of study inclusion (euthymia, Eu) were interviewed only once. At the start of each assessment, a clinician collected clinical demographics, including HDRS and YMRS, and provided an Empatica E4 wristband [34] which participants were required to wear on their non-dominant wrist until the battery ran out (~48 h). A total of 75 subjects, amounting to a total of 149 recording sessions (i.e., over 7000 h), were available at the time of conducting this study. An overview of the cohort clinical-demographic characteristics is given in Table 2 and the number of recordings available per observation time (T0 to T3) by diagnosis is given in Supplementary Figure (SF) 1; observation times (T0 to T3) merely reflect how the data collection campaign was conducted and were not used (or implicitly assumed) as labels for any of the analysis herewith presented. Given the naturalistic study design, medications were prescribed as part of the regular clinical practice: subjects on at least one antidepressant, lithium, an anticonvulsant, or at least one antipsychotic were respectively 37.83%, 70.94%, 34.45%, 12.16% of the cohort. The median (interquartile range) time since disease onset was 6 (14) years.

**Table 2:** Clinical-demographic characteristics of the study population (N = 75).

	MEAN (SD)	MEDIAN (IQR)
AGE	44.66 (14.42)	45.00 (24.50)
HDRS (TOTAL)	7.27 (6.94)	4.00 (6.00)
YMRS (TOTAL)	7.21 (8.75)	3.00 (10.00)
NUMBER OF SUBJECTS (%)		
SEX	male: 33 (44) female: 42 (56)	
MOOD STATE	**MDE-MDD**: 9 (12) **EU-MDD**: 3 (4) **MDE-BD**: 12 (16) **ME**: 28 (37) **MX**: 7 (9) **EU-BD**: 16 (21)	
ASSESSMENT(S)	**1**: 75 (100) **2**: 44 (59) **3**: 22 (29) **4**: 8 (11)	

According to the DSM-5, an MD can be categorized as either a major depressive episode or a manic episode. As a bridge between these two, the DSM-5 admits a mixed symptoms specifier (MX) to cases where symptoms from both polarities are present.

*EU-BD* euthymia in bipolar disorder, *EU-MDD* euthymia in major depressive disorder, *HDRS* Hamilton Depression Rating Scale, *IQR* inter-quartile range, *MDE-BD* major depressive episode in bipolar disorder, *MDE-MDD* major depressive episode in major depressive disorder, *ME* manic episode, *MX* mixed symptoms episode, *SD* standard deviation, *YMRS* Young Mania Rating Scale.

The E4 records the following sensor modalities (we report their acronyms and sampling rates in parentheses): 3D acceleration (ACC, 32 Hz), blood volume pressure (BVP, 64 Hz), electrodermal activity (EDA, 4 Hz), heart rate (HR, 1 Hz), inter-beat interval (IBI, i.e., the time between two consecutive heart ventricular contractions) and skin temperature (TEMP, 1 Hz). IBI was not considered due to extensive sequences of missing values across all recordings. This is likely due to high sensitivity to motion and motion artifacts, as observed previously [35].

### Data pre-processing

An E4 recording session comes as a collection of 1D arrays of sensory modalities. We quality-controlled data to remove physiologically implausible values with the rules by Kleckner et al. [36] and the addition of a rule to remove HR values that exceeded the physiologically plausible range (25–250 bpm). The median percentage of data per recording session discarded from further analyses because of the rules above was 8.05 (range 1.95–32.10). Each quality-controlled recording session was then segmented using a sliding window, whose length (τ, in wall-time seconds) is a hyperparameter, enforcing no overlap between bordering segments (to prevent models from exploiting overlapping motifs between segments). These segments (*x*_*i*_) and the corresponding 28 clinician-scored HDRS/YMRS items (*y*_*i*_) from the subjects wearing the E4 formed our dataset, ${\left\{\left({x}_{i},{y}_{i}\right)\right\}}_{i=1}^{N}$. Note that all segments coming from a given recording session share the same labels, i.e., the HDRS/YMRS scores of the subject wearing the E4. HDRS/YMRS items map symptoms spanning mood, sleep, and psycho-motor activity. Some likely fluctuate over a 48-h session, especially in an ecological setting where treatments can be administered (e.g., Y9 disruptive-aggressive behavior may be sensitive to sedative drugs). To limit this, we isolated segments from the first five hours (*close-to-interview* samples) and used them for the main analysis, splitting them into train, validation, and test sets with a ratio of 70-15-15. Then, to study the effect of distribution shift, we tested the trained model on samples from each 30-min interval following the first five hours of each recording (*far-from-interview* samples). It should be noted that further to a shift in the target variables, a shift in the distribution of physiological data collected with the wearable device is to be expected [37], owing to different patterns of activity during the day, circadian cycles, and administered drugs. Details on the number of recording segments in train, validation, and test splits are given in Supplementary Table (ST) 1.

### Evaluation metrics

HDRS and YMRS items are ordinal variables. For instance, *H11 anxiety somatic* has ranks 0-Absent, 1-Mild, 2-Moderate, 3-Severe, or 4- Incapacitating. The item distribution (see SF2) was imbalanced towards low scores due to patients on an acute episode usually receiving intensive treatment such that acute states tend to be relatively short-lived periods in the overall disease course [30, 31]. This can be quantified with the ratio between the cardinality of the majority rank and that of the minority rank ρ: e.g., say there are 100, 90, 50, 30, and 10 recording segments with an *H11* rank of respectively 0, 1, 2, 3, and 4, then ρ is 100/10 = 10 as 100 is the cardinality of the *H11* rank (0) with the highest number of segments and 10 is the cardinality of the *H11* rank (4) with the lowest number of segments. Metrics accounting for class imbalance should be used when evaluating a classification system in such a setting to penalize trivial solutions, e.g., systems always predicting the majority class in the training set regardless of the input features. We used Cohen’s κ, in particular its quadratic version (QCK), since, further to its suitability to imbalanced ordinal data, it is familiar and easily interpretable to clinicians and psychometrists [38–41]. It expresses the degree to which the ANN learned to score segments in agreement with the clinician’s assessments. This is similar to psychiatric clinical trials where prospective raters are trained to align with assessments made by an established specialist [42]. Cohen’s κ takes values in [−1,1], where 1 (−1) means perfect (dis)agreement. In a psychiatric context, 0.40–0.59 is considered a good range while 0.60–0.79 is a very good range [43]. Cohen’s κ compares the observed agreement between raters to the agreement expected by chance taking into account the class distributions; the quadratic weightage in QCK penalizes disagreements proportionally to their squared distance. As individual HDRS/YMRS items have different distributions (see SF2), we checked whether item level performance was affected by sample Shannon entropy (ℋ). To this end, we computed a simple Pearson correlation coefficient (R) between item QCK and ℋ.

### Model design

**Fig. 2:**
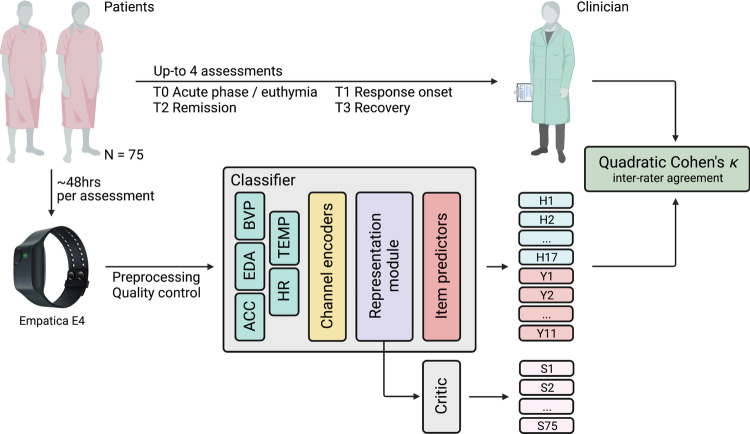
Analysis workflow. Patients had up to four assessments. At the start of each assessment, a clinician scored the patient on the Hamilton Depression Rating Scale (H in the figure) and Young Mania Rating Scale (Y) and provided an Empatica E4 device asking the patient to wear it for ~48 h (i.e., average E4 battery life). An Artificial Neural Network (ANN) model is fed with recording segments and is tasked with recovering clinician scores. The quadratic Cohen’s κ measures the degree to which the machine scores are in agreement with those of the clinician. The ANN model is made of Classifier (CF) and Critic (CR). The former comprises three main modules: (1) Encoder (EN), projecting input sensory channels onto a new space where all channels share the same dimensionality, regardless of the native E4 sampling frequency; (2) Representation Module (RM), extracting a representation h that is shared across all items; and (3) one Item Predictor IP_*j*_ for each item. CR is tasked with telling subjects (S in the figure) apart using *h* and is pitted in an adversarial game against RM(EN(⋅)), designed to encourage the latter to extract subject-invariant representations.

The task at hand is supervised, specifically, we sought to learn a function mapping recording segments to their HDRS and YMRS scores: $f:{x}_{i}\mapsto {\hat{y}}_{i}$. The model we developed to parametrize *f* comprised two independent sub-models (Fig. 2): (a) a **classifier** (${\mathsf{CF}}$), which learns to predict the HDRS/YMRS scores from patients’ physiological data, and (b) a **patient critic** (${\mathsf{CR}}$), which penalize ${\mathsf{CF}}$ for learning subject-specific features (i.e., memorize the patient and their scores), rather than features related to the underlying disorder shared across patients. Both CF and CR are simply compositions of mathematical functions, that is layers of the neural network. The CF module itself consisted of three sequential modules (or, equivalently, functions): (a.1) a *channel encoder* (EN) for projecting sensory modalities onto the same dimensionality regardless of the modality’s native sampling rate so that they could be conveniently concatenated, (a.2) a *representation module* (RM) for extracting features, and lastly, to address **(c1)** multi-task learning, (a.3) 28 parallel (one for each item) *item predictors* (IP), each learning the probability distribution over item ranks conditional on the features extracted with RM. The critic module CR, instead, uses the representation from RM for telling subjects apart. CR competes in an adversarial game against EN and RM, designed to encourage subject-invariant representations. Details on the model’s architecture, the mathematical form of CF and CR, and the model’s loss are given in “Supplementary Methods – Model architecture and loss functions”.

### Learning from imbalanced data

We adapted to our use case the following three popular imbalance learning approaches. (i) Focal loss [44]: the categorical cross-entropy (CCE) loss from the item predictor IP_*j*_ was multiplied during training by a scaling factor correcting for rank frequency (such that under-represented ranks have a similar weight on the loss as over-represented ones) while at the same item focusing on instances where the model assigns a high probability to the wrong rank (these are instances the model is very confident about but its confidence it misplaced as it is outputting the wrong rank). (ii) Probability thresholding [45]: during inference, probabilistic predictions for each rank under the jtℎ item were divided by the corresponding rank frequency (computed on the training set), plus a small term to avoid division by zero in case of zero frequency ranks. The new values were then normalized by the total sum. (iii) Re-sampling and loss re-weighting: HDRS/YMRS severity bins (defined in [33]) were used to derive a label which was then used to either random under-sampling (RUS) or random over-sampling (ROS) segments with, respectively, over-represented and under-represented labels. The loss of *x*_*i*_ was then re-scaled proportionally to the re-sampling ratio of its class.

### Hyperparameter tuning

In order to find the hyperparameters that yield the best QCK in the validation set, we performed an exhaustive search using Hyperband Bayesian optimization [46]. ST2 shows the hyperparameters search space and the configuration of the best model after 300 iterations. We also computed which hyperparameters were the best predictors of the validation QCK. This was obtained by training a random forest with the hyperparameters as inputs and the metric as the target output and deriving the feature importance values for the random forest. Details on model training are given in “Supplementary Methods – Model training”.

### Baseline model using classical machine learning

Most previous works into personal sensing for MDs (as discussed in the Introduction) did not use deep learning for automatically learning features from minimally processed data but deployed classical ML models relying on hand-crafted features. Thus, we developed a baseline in the same spirit, in order to better contextualize our deep-learning pipeline performance on *close-to-interview* samples. Namely, from the same recording segments inputted to the ANN we extracted features (e.g., heart rate variability, entropy of movement) with a commonly used feature extractor for Empatica E4, named FLIRT [47], and developed random forest classifiers (28 in total, as many as there are HDRS and YMRS items), using random oversampling to handle class. We opted for random forest since it was a popular choice in previous relevant works [22, 27]. The hyperparameter space was explored with a random search of 300 iterations for each classifier. Details are given in ST3.

### Prediction error examination

Towards gaining insights into the best-performing setting among those explored in the experiments detailed above we computed residuals on *close-to-interview* samples and illustrated their distribution across items. For the sake of better comparability, items with a rank step of two (e.g., *Y5 irritability*) were re-scaled to have a rank step of one like other items. Furthermore, towards investigating correlations between residuals, checking for any remarkable pattern in view of the natural correlation structure of HDRS and YMRS, we estimated a regularized partial correlation network, in particular a Gaussian graphical lasso (glasso [48]), over item residuals (“Supplementary Methods – Gaussian Graphical Lasso” for details). Lastly, towards having a subject-level perspective, we computed the item-average macro-averaged F1 score (F1^M^) for each subject, checked for any pattern of cross-subjects variability in subject performance, and checked for association with available clinical-demographic variables (age, sex, HDRS/YMRS total score) using Pearson’s R and independent samples t-test with Bonferroni correction.

### Channels importance

In order to assess each sensory modality contribution to the HDRS-YMRS items prediction, we took a simple, model-agnostic approach to assess each individual channel contribution to the task at hand. That is to say, we selected the system performing best on the task and re-trained it including all channels (tri-axial ACC, EDA, BVP, HR, and TEMP) but one. For each left-out channel, we measured the difference in performance across items relative to the baseline model (the one trained on all channels).

## Results

### Best model details – ANN

The loss type is the hyperparameter most predictive of validation QCK (ST4). The selected model employs the Cohen’s κ loss with quadratic weightage [39] (**c2**). The best model uses a (small) critic penalty (λ = 0.07) added to the main objective, i.e., scoring HDRS/YMRS (**c3**). However, the training curve shows that the reduction in the multi-task loss (each item prediction can be thought of as a task) across epochs is paralleled by the reduction in the loss (cross-entropy) paid by CR, tasked with telling subjects apart. Resampling and loss re-weighting (**c4**) is the preferred strategy for class imbalance. We found that a segment length of 16 s yields the best result. The difference in QCK (Δ_QCK_) for other choices of τ (in seconds) relative to the best configuration is −0.092 (8 s), −0.100 (32 s), −0.191 (64 s), −0.246 (128 s), 0.355 (256 s), −0.4431 (512 s), −0.577 (1024 s). Note that τ was explored among powers of 2 for computational convenience and that, when segmenting the first 5 hours of each recording, different τ values produced different sample numbers and lengths (the lower the τ values, the higher the number of samples, the shorter the sample). The predictive value of hyperparameter τ towards validation QCK is fairly low relative to other hyperparameters.

### Main results

Our best ANN model achieves an average QCK across HDRS and YMRS items of 0.609 in *close-to-interview* samples, a value that can be semi-qualitatively interpreted as moderate agreement [49], confidently outperforming our baseline random forest model that only reached an average QCK of 0.214. Item level QCK correlates weakly (*R* = 0.08) with the degree of item class imbalance (ρ) but fairly (*R* = 0.42) with item ℋ. Table 3a shows QCK for each item in HDRS and YMRS. Briefly, QCK is highest for *H12 somatic symptoms gastrointestinal* (0.775) and lowest for *H10 anxiety psychic* (0.492). *H10* has also the highest ℋ (1.370), however, *H7 work and activities*, despite having the second highest ℋ (1.213), has a QCK of 0.629, ranking as the 9^th^ best-predicted item.

**Table 3:** (a) Quadratic Cohen’s κ ranges from 0.775 on “somatic symptoms gastrointestinal” and to 0.492 on “anxiety psychic” (mean of 0.609). (b) Quadratic Cohen’s *κ* deteriorated across both Hamilton Depression Rating Scale (HDRS) and Young Mania Rating Scale (YMRS) on segments taken further away from when the interview took place.

(a)										
Item QCK	H1 0.642	H2 0.624	H3 0.694	H4 0.534	H5 0.595	H6 0.512	H7 0.629	H8 0.604	H9 0.508	H10 0.492
	H11 0.636	H12 0.775	H13 0.582	H14 0.594	H15 0.691	H16 0.637	H17 0.574	Y1 0.602	Y2 0.590	Y3 0.627
	Y4 0.629	Y5 0.591	Y6 0.572	Y7 0.582	Y8 0.588	Y9 0.755	Y10 0.602	Y11 0.566		

(b)							
Item-average QCK	5:01–6:00	6:01–6:30	6:31–7:00	7:01–7:30	7:31–8:00	8:01–8:30	8:31–9:00
HDRS	0.483	0.301	0.183	0.178	0.180	0.177	0.178
YMRS	0.499	0.307	0.182	0.181	0.181	0.173	0.175

Notes for (a): Item level QCK across HDRS and YMRS items. See Supplementary Table 5 for macro-averaged F1 scores.

Notes for (b): Item average QCK is herewith shown,see Supplementary Fig. 3 for a zoom on individual items across all available 30-min intervals.

### Shift over time

When tested on *far-from-interview* samples, our system overall has a drop in performance (Table 3b and SF3). The average QCK is 0.498, 0.303, and 0.182 on segments taken respectively from the first, second, and third thirty-minute intervals. Thereafter, it fluctuates through the following thirty-minute intervals with 0.061 as the lowest value 15 h into the recording. The items with the biggest drop in QCK relative to their baseline value across the first three 30-min intervals are *H9 agitation*, *H10 anxiety somatic*, *Y4 sleep*, and *Y9 disruptive-aggressive behavior*. On the other hand, items that retain their original QCK value the most in the first three 30-min intervals are *H1 depressed mood*, *Y11 insight*, *H2 feelings of guilt*, and *H17 insight*. This pattern matches clinical intuition as items in the former group may be more volatile and reactive to environmental factors (including medications), whereas items in the latter group tend to change more slowly.

### Post-hoc diagnostics

In order to gain further insights into the errors that our system made on *close-to-interview* holdout samples, we studied the distribution of residuals, i.e., the signed difference between prediction $\hat{y}$ and ground truth $y$. SF4 illustrates that the model is correct most of the time, residuals are in general evenly distributed around zero, and when wrong the model is most often off by just one item rank. Summing individual items’ predictions, we could get predictions on HDRS/YMRS total sore (which is indeed simply the sum over the questionnaire items) which had a Root Mean Squared Error (RMSE) of 4.592 and 5.854, respectively.

Furthermore, we investigated the correlation structure among item residuals to check whether any meaningful pattern emerged. SF5 shows the undirected graphical model for the estimated probability distribution over HDRS and YMRS item residuals. The graph only has positive edges, that is, only positive partial correlations between item residuals and co-variates. HDRS and YMRS nodes tend to have weak interactions across the two scales, with the exception of nodes that map the same symptom, e.g., *Y11* and *H17* both query *insight*. Within each scale, partial correlations are stronger among nodes underlying a common symptom domain, e.g., *H1* and *H2* constitute “core symptoms of depression” [50], and speech (*Y6*) is highly related to mood (*Y1*) and thought (*Y7*, *Y8*) [51]. Average node predictability for HDRS and YMRS items, a measure of how well a node can be predicted by nodes it shares an edge with, akin to R^2^, is 48.43%. Stability analyses showed that some edges are estimated reliably (i.e., they were included in all or nearly 500 bootstrapped samples), but there also is considerable variability in the edge parameters across the bootstrapped models. Subjects’ item average F1 macro-averaged F1 score (F1^M^) score had a mean value of 0.605 (std = 0.015) with no subjects standing out for a remarkable high (or low) performance. No associations with age, sex, HDRS/YMRS total score emerged (SF6).

### Channels contribution

**Fig. 3:**
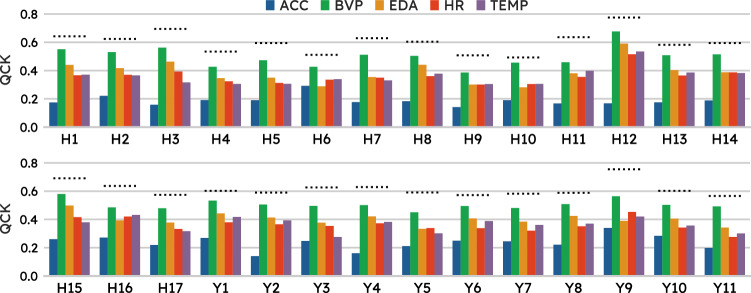
All physiological modalities contributed to the test performance across items, however, this was particularly pronounced for Acceleration (ACC) and relatively modest for Blood Volume Pressure (BVP). Effect of dropping individual channels on item performance. The dotted line is at the level of baseline model performance while each bar indicates the performance upon re-training the best model including all channels but the one corresponding to the bar color code, as shown in the legend.

We were interested in whether physiological modalities contributed differently towards performance across items. This question, further to clinical interest, has also practical implications since other devices may not implement the same sensors as Empatica E4. Figure 3 shows that while all modalities seem to positively contribute to test performance across items, this is markedly the case with ACC as the model records the biggest drop in performance upon removal of this channel from input features. Specifically, upon zeroing out the contribution of ACC, the biggest deterioration in performance was observed for items mapping anxiety (e.g., *H11 anxiety somatic* Δ_QCK_ = − 0.321), *YMRS4 sleep*, and *YMRS9 disruptive- aggressive behavior* (with a Δ_QCK_ of −0.371 and −0.281 respectively), and core depression items (e.g., *H1 depressed mood* Δ_QCK_ = − 0.276). On the other hand, the contribution of BVP was relatively modest since, upon dropping this channel, items generally had only a marginal reduction in QCK.

## Discussion

In this work, we proposed a new treatment of MDs monitoring with personal sensing: inferring all 28 items from HDRS and YMRS, the most widely used clinician-administered scales for depression and mania respectively. Casting this problem as a single-label prediction, e.g., disease status or the total score on a psychometric scale, as done previously in the literature, dismisses the clinical complexity of MDs, thereby losing actionable clinical information, which is conversely preserved in the task we introduced here. Furthermore, the predicted total score on a psychometric scale can always be recovered if item-level predictions are available by simply summing them out, whereas the other direction, i.e., going from total score to individual item predictions, is not possible.

We developed and tested our framework with samples taken over five hours since the start of the clinical interview (*close-to-interview* samples), achieving moderate agreement [52] with expert clinician (average QCK of 0.609) on a holdout set and showing that our deep-learning pipeline vastly exceeded the performance (average QCK of 0.214) of traditional ML baseline relying on hand-crafted features. Item level performance showed a fair correlation with item ℋ, indicating that items with a higher “uncertainty” in their sample distribution tend to be harder to predict. The difference in ℋ is partly inherent to the scale design, as different items admit a different number of ranks. HDRS/YMRS total scores, with the range of [0–52] and [0–60], were predicted with an RMSE of 4.592 and 5.854, respectively (note that item level error compounds across items when summing them out). A five and three-point interval are the smallest bin widths for YMRS and HDRS respectively [53, 54], e.g., a YMRS total score in the range of [20–25] is considered a mild mania and an HDRS total score in [19–22] is considered as severe depression. This shows that on average our model would be off by two score bands at most, in case of a true score falling on the edge of a tight severity bin (i.e., the ones reported above). We recommend caution in interpreting these results however as metrics suited for continuous target variables, unlike QCK and F1^M^, are not robust in settings where the distribution is skewed (towards lower values in our case). Furthermore, while these results are comparable to previous ones (e.g., Ghandeharioun et al. [23] reported a RMSE of 4.5 on the HDRS total score), differences in the sample limit any direct comparison.

When used on samples collected from thirty-minute sequences following the first five hours of the recordings (*far-from-interview* samples), our model had a significantly lower performance with average QCK declining down to 0.182 in the third half-hour and then oscillating but never recovering to the original level. Consistently with clinical intuition, items suffering the sharpest decline relative to their baseline performance were those mapping symptoms that naturally have a higher degree of volatility (e.g., *H9 agitation*) while items corresponding to more stable symptoms (e.g., *H17 insight*) had a gentler drop in performance. Besides (some) symptoms plausibly changing over time, a shift in the physiological data distribution is very likely in a naturalist setting.

Residuals on holdout *close-to-interview* samples showed a symmetric distribution, centered around zero, thus the model was not systematically predicting either over- or under-predicting. The network of item residuals illustrated that our model erred along the correlation structure of the two symptom scales, as stronger connections were observed among items mapping the same symptom or a common domain. An ablation study over input channels showed that ACC was the most important modality, lending further support to the discriminative role of actigraphy with respect to different mood states [14]. Coherently, items whose QCK deteriorated the most upon removing this channel were those mapping symptom domains clinically observable through patterns of motor behavior.

In conclusion, we introduced a new task in personal sensing for MDs monitoring, overcoming limitations of previous endeavors which reduced MDs to a single number, with a loss of actionable clinical information. We indeed advocate for inferring symptoms’ severity as scored by a clinician with the Hamilton Depression Rating Scale-17 [5] (HDRS) and the YMRS [6]. We developed a deep learning pipeline inputting physiological data recordings from a wearable device and outputting HDRS and YMRS scores in substantial agreement with those issued by a specialist (QCK = 0.609). This outperformed a competitive classical machine learning algorithm. We illustrated the main machine learning challenges associated with this new task and pointed to generalization across time as our key area of future research.

### Limitations

We would like to highlight several limitations in our study. (a) All patients were scored on HDRS and YMRS by the same clinician. However, having scores from multiple (independent) clinicians on the same patients would help appreciate model performance in view of inter-rater agreement. (b) The lack of follow-up HDRS and YMRS scores within the same session did not allow us to estimate to what degree a shift in target variables might be at play. Relatedly, we acknowledge that the choice of five hours for our main analyses may be disputable and other choices may have been valid too. Five hours was an informed attempt to trade off a reasonably high number of samples with a minimal distribution shift over both target variables and physiological data; studying the effect of different cut-offs was not within the scope of this work. (c) Given the naturalist setting, medications were allowed, and their interference could not be ruled out. (d) As pointed out by Chekroud et al. [52], the generalizability of AI systems in healthcare remains a significant challenge. While we tested our method on out-of-distribution samples explicitly (close-to-interview vs far-from-interview), other aspects of generalization that are meaningful to personal sensing, such as inter-individual and intra-individual performance, have not yet been tested. For instance, we evaluated our methods on data obtained in a single centre, and it is unclear how well the model would perform in a cross-clinic setting.

### Future work

(i) The decline in performance over future time points stands out as the main challenge towards real-world implementations and suggests that the model struggles to adapt to changes in background (latent) variables, e.g., changes in activity patterns. Research into domain adaptation should therefore be prioritized. We also speculate that MD symptoms and relevant physiological signals have slow- as well as fast-changing components. A segment length of 16 s would seem unsuitable for representing the former and an attempt should be made at capturing both. (ii) Generalization of unseen patients is a desirable property in real-world applications and something we consider exploring in the future. Another approach to tackle this point is to develop (or fine-tune) a model for each individual patient, as done in related fields [55]. (iii) Supervised learning systems notoriously require vast amounts of labeled data for training; as annotation (i.e., enlisting mental health specialists to assess individuals and assign them diagnoses and symptoms’ severity scores) is a major bottleneck in mental healthcare [56], self-supervised learning [57] should be considered for applications using the E4 device. (iv) For an ML system to be trustworthy and actionable in a clinical setting, further research into model explainability and uncertainty quantification is warranted [58].

### Supplementary information

Supplementary Material

## Data Availability

The software codebase used is available at https://github.com/april-tools/wear-your-scales. Python 3.10 programming language was used for the symptoms scoring system, where deep learning models were implemented in PyTorch [59], hyperparameter tuning and visualization model performance were performed in Weights and Biases [60], and random forest classifiers were developed in scikit-learn [61]. Graphical modeling of the residuals and related analyses were performed in R 4.2.2 using packages *qgraph* [62] for network estimation and visualization, and *bootnet* [63] for bootstrapping. Data in de-identified form may be made available from the corresponding author upon reasonable request.
