# Supplementary material for: Automated mood disorder symptoms monitoring from multivariate time-series sensory data: getting the full picture beyond a single number
Source: Transl Psychiatry. 2024 Mar 26;14:161. doi: 10.1038/s41398-024-02876-1 (PMC10965916; doi:10.1038/s41398-024-02876-1)
Supplement: Supplementary file 1 — Supplementary Material [file 41398_2024_2876_MOESM1_ESM.docx]

Supplementary Table 1. **Number of recording segments across train, validation, and test splits by segment length.** While the same parts of the recording were used for train, validation, and test sets, regardless of the choice of segment length τ, different 𝜏 values (in seconds) resulted in different numbers of segments as shown below. While exploring different values of 𝜏 might add a layer of complexity to data splits, yet this was more principled than simply fixing segment length to an arbitrary number as done in previous works.

| **Segment length** 𝝉 **(sec)** | **Train (N)** | **Validation (N)** | **Test (N)** |
| --- | --- | --- | --- |
| 8 | 232894 | 49905 | 49905 |
| 32 | 58224 | 12476 | 12476 |
| 64 | 29112 | 6238 | 6238 |
| 128 | 14608 | 3130 | 3130 |
| 256 | 7252 | 1554 | 1554 |
| 512 | 3528 | 735 | 735 |
| 1024 | 1728 | 432 | 432 |

# Supplementary Table 2. Artificial Neural Network hyperparameter search space and final configuration after Hyper-band Bayesian optimization.

| **Hyperparameter** | **Search Space** | **Final Value** |
| --- | --- | --- |
| Batch size | Uniform, min: 8, max 128, interval: 8 | 96 |
| Critic 𝜆 | Uniform, min: 0, max 1 | 0.0723 |
| Segment length 𝜏 | Uniform, 2^n^, 3 ≤ n ≤ 10 | 16 |
| Focal loss 𝛾 | 0, 1, 2, 3, 4, 5 | 5 |
| Imbalance mode | N/A, Focal loss, prob. threshold, resample and reweight | Focal loss |
| Learning rate 𝛼_lr_ | Uniform, min: 0.0001, max: 0.01 | 0.0009 |
| Preprocessing | N/A, Normalization, Standardization | Standardization |
| Loss function | Cross-entropy, Weighted 𝜅, ONTRAM | Weighted 𝜅 |
| Weight decay | Uniform, min: 0, max: 1 | 0.1853 |
| Channel encoders 𝐸𝘕 | | |
| Embedding type | MLP, GRU, Time2Vec | MLP |
| Embedding Dim. | Uniform, min: 32, max: 64, interval: 8 | 312 |
| Representation Module 𝑅𝘔 | | |
| Num. Units | Uniform, min: 8, max: 2048, interval: 8 | 1568 |
| Dropout | Uniform, min: 0, max: 1 | 0.4870 |

Supplementary Table 3. **Random Forest Classifiers search space.** 28 random forest classifiers were developed, one for each HDRS and YMRS item. Hyperparameters for each classifier were selected based on best validation set performance out of 300 randomly sampled configurations.

| **Hyperparameter** | **Search Space** |
| --- | --- |
| Segment length 𝜏 | Uniform, 2^n^, 3 ≤ n ≤ 10 |
| Criterion | Uniform: Gini, entropy log loss |
| Max features | Uniform, min: 8, max 184, interval: 8 |
| Max depth | Uniform: 3, 9, 15, 30, None |
| Num. estimators | Uniform, 10^n^, 2 ≤ n ≤ 5 |

Supplementary Table 4. **Hyperparameters sorted by their importance towards predicting the monitored metric, validation Quadratic Cohen's** 𝛋 **(QCK).** QCK predictability from hyperparameters is derived by training a random forest with the hyperparameters as inputs and the metric as the target output and estimating feature importance values for the random forest. Details at docs.wandb.ai/parameter-importance.

| **Hyperparameter importance** |
| --- |
| loss function 0.205 |
| dropout 0.073 |
| batch size 0.070 |
| weight decay 0.055 |
| preprocessing 0.035 |
| RM num. units 0.033 |
| EN dim. 0.032 |
| critic 𝜆 0.026 |
| learning rate 𝛼_lr_ 0.025 |
| imbalance mode 0.022 |
| focal loss 𝛾 0.019 |
| segment length 𝜏 0.013 |

Supplementary Table 5. **F1 score ranges from 0.877 on “disruptive aggressive behavior” and to 0.379 on “anxiety psychic” with an average of 0.609**. Item level macro-averaged F1 score (F1^M^) across HDRS and YMRS items.

| Item F1^M^ | H1 0.512 | H2 0.531 | H3 0.445 | H4 0.643 | H5 0.702 | H6 0.645 | H7 0.610 | H8 0.398 | H9 0.562 | H10 0.380 |
| --- | --- | --- | --- | --- | --- | --- | --- | --- | --- | --- |
|  | H11  0.560 | H12  0.860 | H13  0.723 | H14  0.641 | H15  0.753 | H16  0.736 | H17  0.681 | Y1  0.629 | Y2  0.599 | Y3  0.481 |
|  | Y4  0.647 | Y5  0.710 | Y6  0.581 | Y7  0.501 | Y8  0.562 | Y9  0.877 | Y10  0.653 | Y11  0.430 |  | |

Supplementary Figure 1. **Number of recording sessions available at different time points (T0-T3) by diagnosis.** A total of 149 recordings were available from the data collection campaign at the moment of conducting this study. Mood disorders manifest in two polarities, mania, and depression. Major Depressive Disorder (MDD) is characterized by Major Depressive Episodes (MDEs) only, whereas Bipolar Disorder (BD) features (hypo)manic episodes (ME) that can alternate with MDEs. The presence of symptoms from both polarities within the same episode connotes a mixed episode (MX). Patients with a former mood disorder diagnosis, clinically stable at present are said to be Euthymic (Eu). With the exception of Healthy Controls (HCs) and Euthymic Patients, other subjects are recruited at the onset of a disease episode. They are assessed at subsequent stages (maximum four) during their clinical course. Exclusion criteria are co-morbidity with another psychiatric or neurological disorder or current drug abuse and pregnancy.


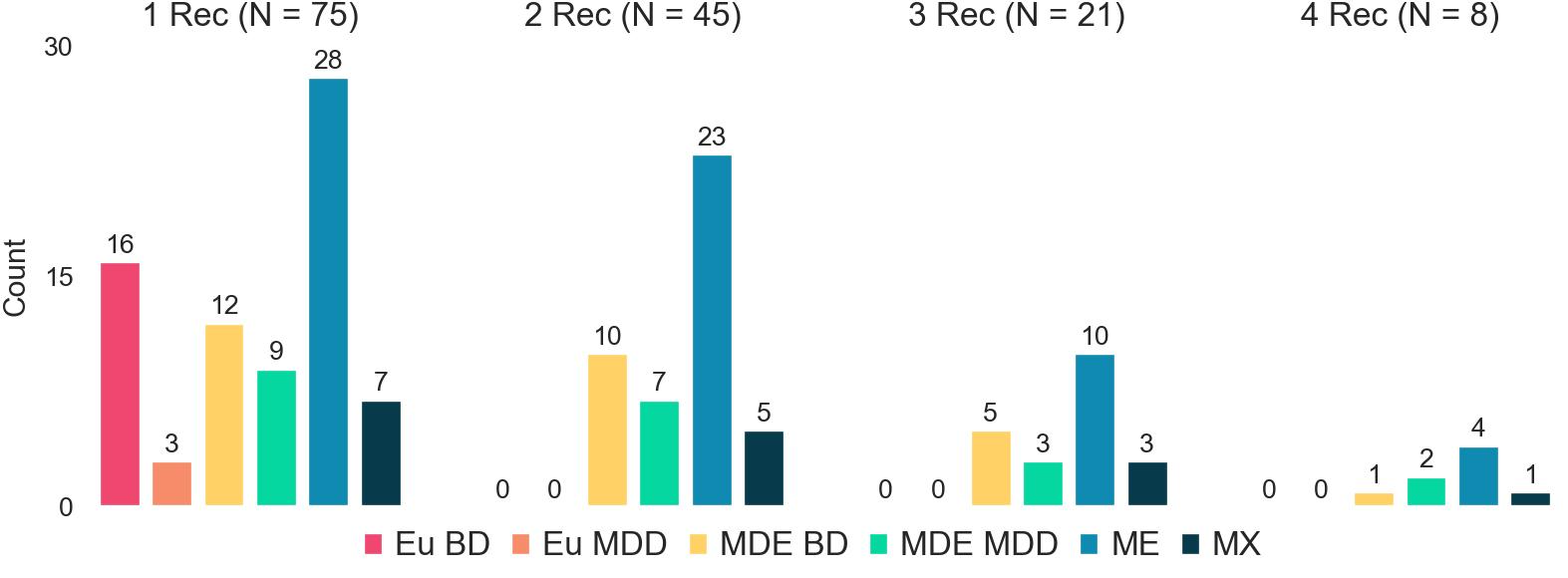


Supplementary Figure 2. **Distribution over Hamilton Depression Rating Scale (blue) and Young Mania Rating Scale (red) items across the recording sessions used in this study.** The number above each bar plot, ρ, is the cardinality of the majority class over that of the minority rank. Higher values of ρ thus indicate a more pronounced imbalance between the majority and the minority rank.


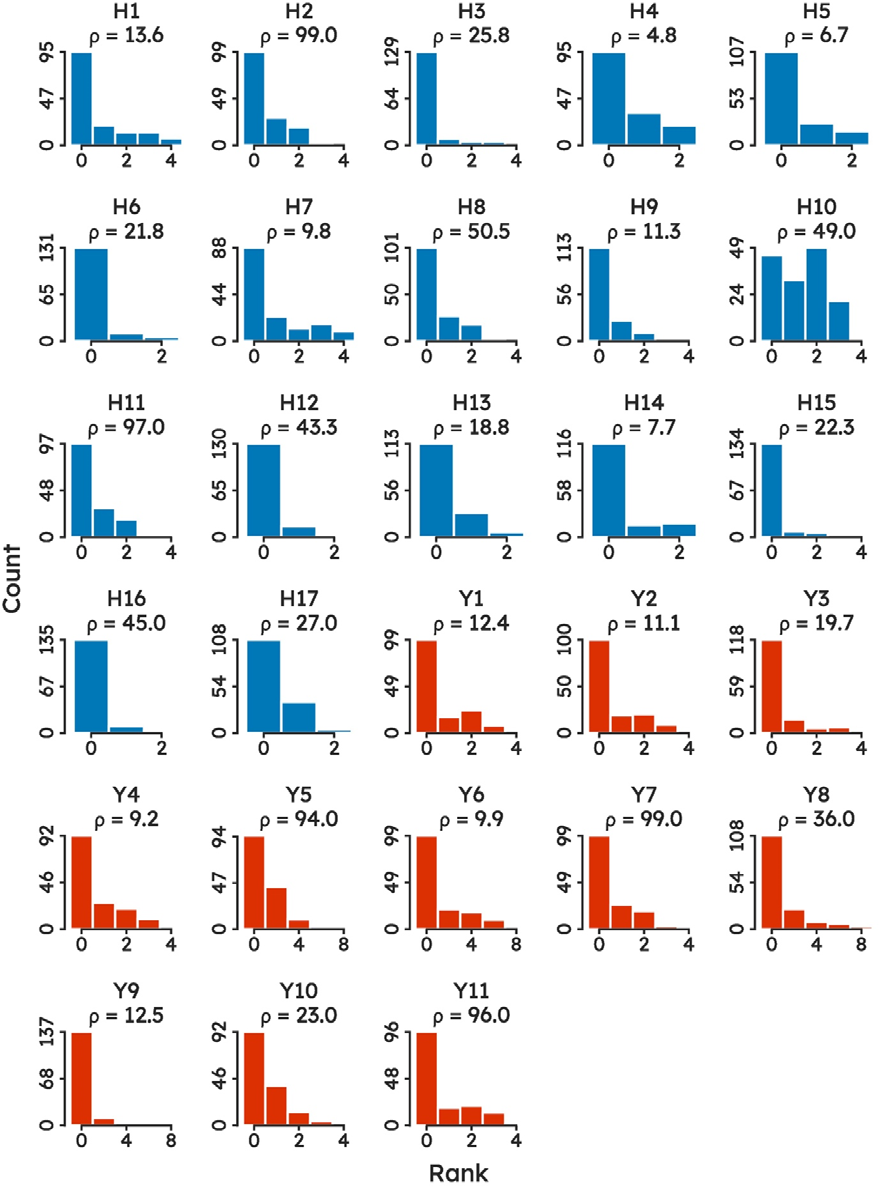


Supplementary Figure 3. **Quadratic Cohen’s κ (QCK) deteriorated across all items when the model was tested on segments taken further away from when the interview took place.** The first point (0 on the x-axis) is the baseline performance, i.e. holdout segments from the first five hours of recordings (**close-to-interview**). The following points refer to the successive thirty-minute intervals (**close-to-interview**).


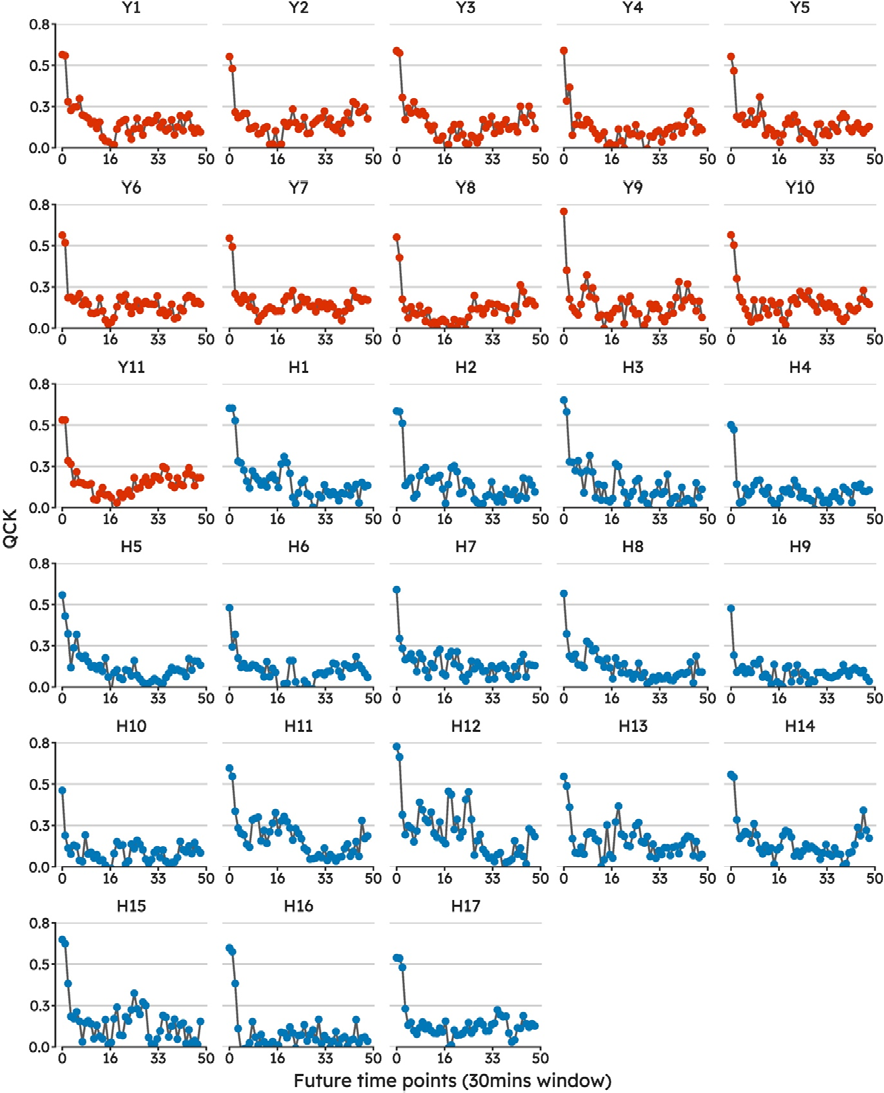


Supplementary Figure 4. **Item residuals overall show a symmetric distribution centered around zero, showing that the model is correct most of the time, it is not systematically either under- or over-predicting, and when wrong, it is usually off by only one.** Residuals, signed difference between prediction ($\hat{y}_{i}$) and ground truth (𝑦), are shown across Hamilton Depression Rating Scale (Top) and Young Mania Rating Scale (Bottom) items.


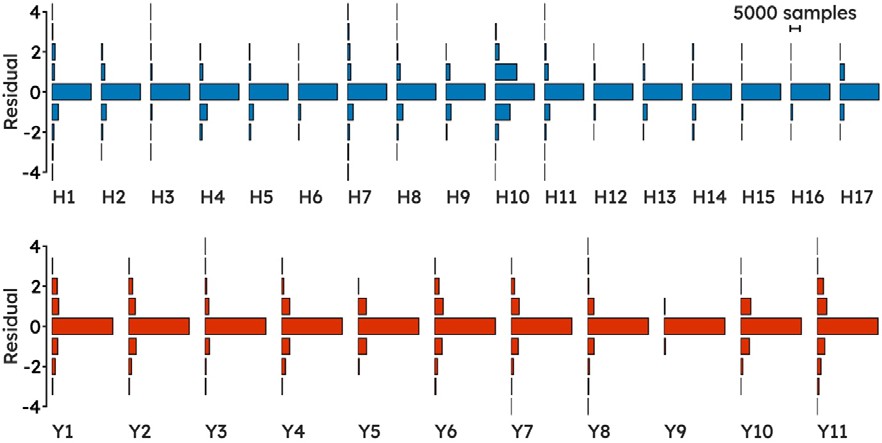


Supplementary Figure 5. **Partial correlations between item residuals indicate that the model learned the scales’ natural correlation structure.** Network displaying the relationship between HDRS (blue) and YMRS (red) item residuals. Green edges represent positive partial correlations between variables. Rings around nodes represent variance in a given variable with shadowed parts displaying the proportion of variance in that node that is explained by nodes that connect with it.


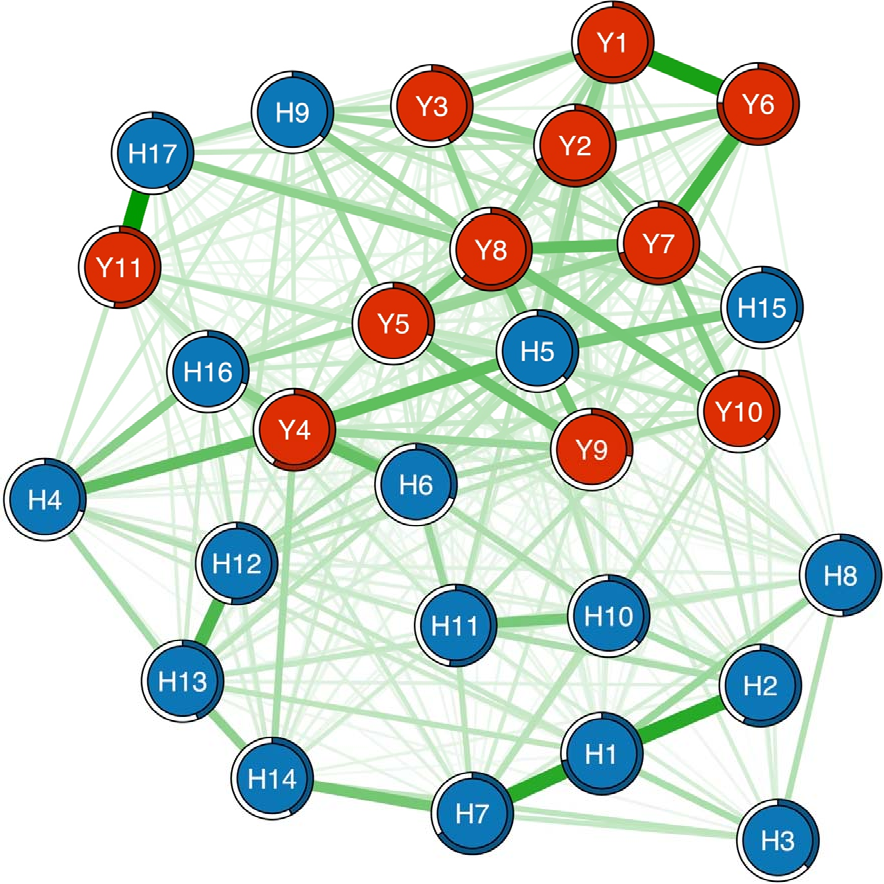


# Supplementary Figure 6. Performance was consistent across subjects, with no associations with age, sex, or total score on psychometric scales. Each point on the scatter plot corresponds to a subject score as expressed with mean item macro-average F1 score (F1^M^). Subjects are sorted in descending order by F1^M^ and assigned a dummy ID for the sake of this plot. We could not reject the null hypothesis that the distributions of item-average F1^M^ scores and, on the other hand, age/HDRS/YMRS underlying the samples are uncorrelated (Pearson R=0.016, p-val_B_=3.762, Pearson R=0.117, p-val_B_=3.964 for HDRS total score, Pearson R=0.152, p-val_B_=3.114 for YMRS total score) or the null hypothesis that difference of item-average F1^M^ mean across males and females is zero (t=1.422, p-val_B_=0.631).

# *
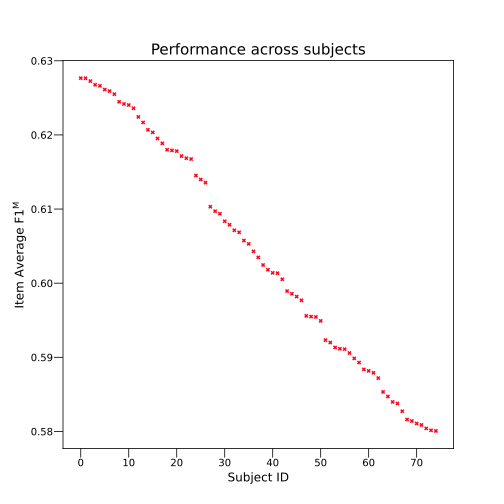
*

# Supplementary Methods

1. **Model architecture and loss functions**

Our Artificial Neural Network (ANN) classifier (a), tasked with inferring Hamilton Depression Rating Scale (HDRS) and Young Mania Rating Scale (YMRS) items, consisted of three modules: channel encoders, representation modules, and item predictors. An auxiliary critic (b) aided the classifier in learning (**challenge c3**) subject invariant representations.

1. **Classifier**

The task of inferring HDRS and YMRS items is an instance of MTL (challenge c1), to which we adopted a hard parameter-sharing approach: all tasks shared the same model trunk $\mathsf{RM}(\mathsf{EN}(\cdot))$, and thus the same base representation of the input data $h_{i}=\mathsf{RM}\left( \mathsf{EN}\left( x_{i} \right) \right)$, which was then distributed across task-specific layers, $\mathsf{IP} ADDIN ZOTERO\_TEMP$^1^. As usually done with hard parameter-sharing^2^, the multi-task loss was set equal to the average of task-specific losses, each weighted by the corresponding item rank step to account for different item weights on the scale total score:

$$\mathcal{L}_{\mathsf{MT}}\left( x_{i};\xi,\phi,\psi\right)=-\frac{r_{j}}{\sum_{j} r_{j}}\mathcal{l}_{j}\left( \mathsf{IP}_{j}(\mathsf{RM}(\mathsf{EN}(x_{i}))),y_{i} \right)$$

where $r_{j}$ is the rank step size of the $j^{th}$item (e.g., Y5 irritability ranks have a step size of 2) and ξ, φ, and ψ are respectively $\mathsf{EN}$, $\mathsf{RM}$, and $\mathsf{IP}$ parameters. Details on the specific form of $\mathcal{l}$, the task-loss, are given below.

**Channel encoders** Since the sampling rate varies across the recorded signals within a segment, these are typically time-aligned, e.g. to the level of a second in wall-time usually via max-pooling or averaging^3,4^, before being further analysed. However, we took a different approach that did not pre-specify the function for time alignment and mapped each channel to the same dimensionality with the use of a channel encoder $\mathsf{EN}$. We experimented with a simple Multilayer Perceptron (MLP), a Gated Recurrent Unit (GRU^5^) or, alternatively, the Time2Vec representation proposed in^6^. Before passing segments through $\mathsf{EN}$, each channel was re-scaled. The optimal embedding dimensionality and re-scaling type (either standardization or normalization) were set during tuning.

**Representation module** We used a BiLSTM^7^ as architecture for our RM, as it belongs to Recurrent Neural Networks (RNNs), a class of deep learning architectures specifically engineered to exploit dependencies in time series data, and, thanks to its bidirectionality in consuming an input sequence, it enjoys a richer time representation than a vanilla RNN.

**Item predictors (challenge c2)** The extracted representation $h=\mathsf{RM}\left( \mathsf{EN}\left( \cdot\right) \right)$ was then used as input to 28 $\mathsf{IP}$, each dedicated to a specific HDRS/YMRS item. We experimented with three different treatments of the target variables, each translating to a different set-up of the task-specific layer and the task-specific loss. 1) Each item score prediction was simply treated as a multi-class classification problem. Accordingly, the $j^{th}$ item predictor consisted of a fully connected layer, with as many output units as the number of ranks under that item, to which a SoftMax activation was applied, and the categorical cross-entropy (CCE) was used as loss function. 2) We used the same task-specific architecture as in 1) but adopted the QWK loss, as proposed in^8^, which re-writes Cohen’s κ in terms of probability distributions. 3) We implemented the ordinal neural network transformation model (ONTRAM^9^) which parameterizes the CCE loss to incorporate the order of the outcome, by deriving class probabilities from the conditional density function of a latent variable onto which observed classes are mapped.

1. **Critic**

We encouraged cross-subjects invariance in the representation extracted with $\mathsf{RM}\left( \mathsf{EN}\left( \cdot\right) \right)$ by adding a critic $\mathsf{CR}$, whose task was to correctly distinguish subjects apart from extracted representation, in an adversarial game, similarly to ^10^ and ^11^. Concretely, $\mathsf{CR}$, a simple MLP, inputs the extracted representation h and is trained to identify subjects from it. $\mathsf{CR}$'s task was therefore to minimize, with respect to $\mathsf{CR}$'s parameters θ, the following CCE loss:

$$\mathcal{L}_{\mathsf{CR}}\left( \mathsf{RM}\left( \mathsf{EN}\left( x_{i} \right) \right);\theta\right)=-\mathbb{1}_{s}\log\mathsf{CR}_{s}\left( \mathsf{RM}\left( \mathsf{EN}\left( x_{i} \right) \right) \right)$$

where $\mathbb{1}_{s}$ is an indicator taking value 1 when the $i^{th}$segments belong to the $s^{th}$ subject and 0 otherwise, and $\mathsf{C}\mathsf{R}_{\mathsf{s}}(\cdot)$ is the critic output (i.e. probabilities from a softmax activation) for the $s^{th}$subject. On their part, $\mathsf{EN}$ and $\mathsf{RM}$ tried to trump the $\mathsf{CR}$by filtering out from $h$ information that could make $\mathsf{CR}$'s task easy, while, at the same time retaining enough useful information for the item predictors $\mathsf{IP}_{j}$. To achieve this, the following term was added to $\mathcal{L}_{MT}$ (the multi-task loss), which was minimized with respect to $\mathsf{EN}$'s and $\mathsf{RM}$'s parameters, ξ and ϕ :

$$\mathcal{L}_{\mathsf{R}}(x_{i};\xi,\phi)=\lambda\left[ -\mathbb{1}_{s}\log(1-\mathsf{CR}_{s}\left( \mathsf{RM}\left( \mathsf{EN}\left( x_{i} \right) \right) \right) \right]$$

where $\lambda\in[0,1]$. The classifier's total loss was then $\mathcal{L}_{\mathsf{CF}}=\mathcal{L}_{\mathsf{MT}}+\mathcal{L}_{\mathsf{R}}$, where $\mathcal{L}_{\mathsf{R}}$ acted as a regulariser, a price $\mathsf{CF}$ paid for encoding subject-specific information in the representation $h$ learned by $\mathsf{RM}(\mathsf{EN}(\cdot))$. Values of $\lambda$ trade off learning cross-subjects invariant representations $h$ against solving the main objective; for $\lambda$ = 0, no incentive is given towards learning cross-subjects invariant representation.

1. **Model training**

All models were trained with AdamW^12^ optimizer for a maximum of 400 epochs. Moreover, to speed up the training and search procedure, we employed an early stopping learning rate scheduler: we reduce the learning rate $\alpha_{\mathsf{lr}}=0.3\alpha_{\mathsf{lr}}$ if the model has not improved in its validation performance after 10 consecutive epochs; we terminate the training procedure if the model has not improved after 2 learning rate reductions. Dropout^13^ and weight decay were added to prevent overfitting.

1. **Gaussian Graphical Lasso**

Network edge sparsity, to avoid false positives, is enforced with the least absolute shrinkage and selection operator (LASSO^14^), which indeed shrinks all edge weights towards zero and sets small weights to exactly zero. The strength of the regularization is traded off by a hyperparameter λ, selected with the Extended Bayesian Information Criterion (EBIC^15^). The EBIC itself has a tuning parameter γ controlling the trade-off between sensitivity and precision, which we set to 0.25 as in ^16^. We also estimated node predictability, measuring how well a node can be predicted by nodes it shares an edge with, which can be interpreted similarly to R^2,17^. Lastly, bootstrapping routines were used to gain insight into the stability of the estimated parameters.

**References**

1 Crawshaw M. Multi-task learning with deep neural networks: A survey. *ArXiv Prepr ArXiv200909796* 2020.

2 Ruder S. An overview of multi-task learning in deep neural networks. *ArXiv Prepr ArXiv170605098* 2017.

3 Adler DA, Wang F, Mohr DC, Choudhury T. Machine learning for passive mental health symptom prediction: Generalization across different longitudinal mobile sensing studies. *Plos One* 2022; **17**: e0266516.

4 Li BM, Corponi F, Anmella G, Mas A, Sanabra M, Hidalgo-Mazzei D *et al.* Inferring mood disorder symptoms from multivariate time-series sensory data. In: *NeurIPS 2022 Workshop on Learning from Time Series for Health*. 2022https://openreview.net/forum?id=awjU8fCDZjS.

5 Chung J, Gulcehre C, Cho K, Bengio Y. Empirical evaluation of gated recurrent neural networks on sequence modeling. *ArXiv Prepr ArXiv14123555* 2014.

6 Kazemi SM, Goel R, Eghbali S, Ramanan J, Sahota J, Thakur S *et al.* Time2vec: Learning a vector representation of time. *ArXiv Prepr ArXiv190705321* 2019.

7 Schuster M, Paliwal KK. Bidirectional recurrent neural networks. *IEEE Trans Signal Process* 1997; **45**: 2673–2681.

8 de La Torre J, Puig D, Valls A. Weighted kappa loss function for multi-class classification of ordinal data in deep learning. *Pattern Recognit Lett* 2018; **105**: 144–154.

9 Kook L, Herzog L, Hothorn T, Dürr O, Sick B. Deep and interpretable regression models for ordinal outcomes. *Pattern Recognit* 2022; **122**: 108263.

10 Özdenizci O, Wang Y, Koike-Akino T, Erdoğmuş D. Learning invariant representations from EEG via adversarial inference. *IEEE Access* 2020; **8**: 27074–27085.

11 Cheng JY, Goh H, Dogrusoz K, Tuzel O, Azemi E. Subject-aware contrastive learning for biosignals. *ArXiv Prepr ArXiv200704871* 2020.

12 Loshchilov I, Hutter F. Decoupled Weight Decay Regularization. In: *International Conference on Learning Representations*. 2019https://openreview.net/forum?id=Bkg6RiCqY7.

13 Srivastava N, Hinton G, Krizhevsky A, Sutskever I, Salakhutdinov R. Dropout: a simple way to prevent neural networks from overfitting. *J Mach Learn Res* 2014; **15**: 1929–1958.

14 Tibshirani R. Regression shrinkage and selection via the lasso. *J R Stat Soc Ser B Methodol* 1996; **58**: 267–288.

15 Foygel R, Drton M. Extended Bayesian information criteria for Gaussian graphical models. *Adv Neural Inf Process Syst* 2010; **23**.

16 Haslbeck J, Waldorp LJ. mgm: Estimating time-varying mixed graphical models in high-dimensional data. *ArXiv Prepr ArXiv151006871* 2015.

17 Haslbeck JM, Waldorp LJ. How well do network models predict observations? On the importance of predictability in network models. *Behav Res Methods* 2018; **50**: 853–861.
